# Efficacy of intranasal non-steroidal anti-inflammatory drugs on pain responses in castrated and tail-docked piglets

**DOI:** 10.1186/s40813-026-00517-1

**Published:** 2026-05-20

**Authors:** Cecília Archangelo Ferreira de Melo, Laya Kannan Silva Alves, Monique Danielle Pairis-Garcia, Juliana Bonin Ferreira, Taylor Brooke Parker, Victoria Rocha Merenda, Kristen Michele Messenger, Pedro Henrique Esteves Trindade

**Affiliations:** 1 Department of Animal Nutrition and Production, School of Veterinary Medicine and Animal Science, University of São Paulo, Pirassununga, SP Brazil; 2 Department of Population Health and Pathobiology, College of Veterinary Medicine, North Carolina State University, Raleigh, NC USA; 3 Department of Biomedical Sciences and Pathobiology, VA-MD College of Veterinary Medicine, Virginia Tech, Blacksburg, VA USA; 4 Department of Large Animal Clinical Sciences, College of Veterinary Medicine, Michigan State University, East Lansing, MI USA

**Keywords:** Animal welfare, Piglet Processing, Analgesia, Cortisol, Pain Scale

## Abstract

Routine piglet processing procedures, such as surgical castration and tail docking, are known to induce pain and stress, and non-steroidal anti-inflammatory drugs (NSAIDs) are commonly administered to mitigate these responses. This study evaluated the effects of processing and NSAID administration on physiological and behavioral indicators of pain and stress in male piglets. A total of 120 piglets (5 days old) were randomly assigned to one of eight treatment groups in a 2 × 4 factorial design, including sham or processed piglets receiving intranasal flunixin meglumine, ketoprofen, meloxicam, or saline solution. Piglets underwent surgical castration and tail docking or sham handling one hour after treatment administration. Plasma cortisol concentrations were assessed at baseline, 1 h, 3 h, and 24 h post-procedure, and pain-related behaviors were evaluated using the UNESP-Botucatu Pig Composite Acute Pain Scale (UPAPS). Processed piglets, both with and without NSAID administration, exhibited higher plasma cortisol concentrations than sham piglets at 1 h post-procedure, indicating an acute stress response, with concentrations returning to baseline by 3 h and 24 h. No treatment effects of NSAIDs were observed for cortisol concentrations. Behavioral assessment revealed higher UPAPS scores in processed piglets at 1 h, 3 h, and 24 h compared with sham piglets, indicating persistent pain-related responses. NSAID treatments did not consistently mitigate behavioral pain expression, although ketoprofen-treated piglets exhibited a transient reduction in UPAPS scores at 3 h post-procedure. Overall, piglet processing elicited acute physiological stress responses and sustained behavioral indicators of pain. These findings support the need for integrated analgesic strategies to more effectively mitigate post-procedural pain and improve piglet welfare.

## Introduction

Piglets are routinely subjected to painful husbandry procedures in the first days of life, commonly referred to as a processing. Piglet processing typically occurs around 3–5 days of age and includes tail docking and surgical castration [1]. Tail docking is performed to reduce the risk of tail biting in group-housed pigs, a damaging behavior that can lead to severe injuries, impair performance, and results in substantial economic losses [2]. Surgical castration of male piglets is primarily carried out to prevent undesirable odors and flavors in pork produced from intact males (i.e. boar taint) and to reduce aggressive behaviors among intact group-housed pigs [3]. In the United States (US), these procedures are commonly performed without analgesia, resulting in acute pain, stress, and inflammatory responses that compromise piglet welfare and productivity [3, 4].

Despite increasing societal concern and scientific advances towards mitigating or eliminating processing pain, no analgesic products are currently approved for use in pigs by the US Food and Drug Administration [5]. This lack of approved products remains a major barrier to the widespread implementation of pain mitigation strategies on commercial production systems [6]. Although non-steroidal anti-inflammatory drugs (NSAIDs) may be administered in an extra-label manner under the supervision of the attending site veterinarian [7], many veterinarians in the US remain reluctant to adopt this practice due to perceived concerns regarding limited or unclear drug efficacy and food safety, despite the availability of data from other countries [6].

Non-steroidal anti-inflammatory drugs are widely used in other livestock species and have shown potential to reduce pain and inflammation in piglets [8]. For instance, Lopez-Soriano et al. [9] reported that transdermal flunixin reduced behavioral indicators of pain assessed using the Unesp-Botucatu Piglet Composite Acute Pain Scale (UPAPS; [10]). Similarly, Nixon et al. [3] observed that different NSAIDs attenuated both physiological (e.g., cortisol concentrations) and behavioral responses following castration and tail docking. Collectively, these findings suggest that NSAIDs may represent an effective option for pain control in commercial swine farms, and when administered via needle-free routes, may also mitigate concerns surrounding food and human safety.

Intranasal administration of analgesic products is currently being investigated as a safe, low-cost alternative route of delivery suitable for large scale commercial production [11]. Pharmacokinetic (PK) studies have demonstrated high bioavailability of intranasal flunixin, with similar Tmax and half-life compared to intramuscular administration [12]. Thus, this route may allow for rapid systemic drug absorption while eliminating the need for injections, thereby reducing stress for piglets and minimizing the risk of human injury. However, despite promising pharmacokinetic results, limited studies have evaluated the efficacy of intranasal administration of flunixin and other NSAIDs for pain mitigation in piglets undergoing painful procedures under commercial conditions. Therefore, the objective of this study was to evaluate the efficacy of flunixin meglumine (F), ketoprofen (K), and meloxicam (M) administered intranasally on mitigating physiological and behavioral indicators of pain in male piglets undergoing processing (castration and tail docking).

## Materials and methods

This study was approved by the Institutional Animal Care and Use Committee of North Carolina State University (IACUC protocol # 23–198).

### Housing and animals

This experiment was conducted on a commercial sow farm in the Southeastern US. Sows and piglets were housed in individual farrowing crates, fully slatted, in tunnel-ventilated, farrowing rooms maintained at an average temperature of 22º ± 1.0 °C. Temperature and ventilation were controlled using a computerized system. Each farrowing crate measured 2.5 m × 0.7 m, with an additional piglet area (2.5 m × 1.3 m) accessible only to piglets. Heat mats were provided for piglets and maintained at approximately 30–35 °C. Lighting was provided from 0600 to 1630 h. Sows were provided *ad libitum* access to water and feed throughout the study. Piglets had continuous access to the sow and to water via a nipple drinker.

Animal care and handling followed the Guide for the Care and Use of Agricultural Animals in Research and Teaching [13]. Surgical castration and tail docking are routine farm practices; therefore, piglets enrolled in this study were not processed solely for research purposes.

### Experimental design and treatment administration

**Fig. 1:**
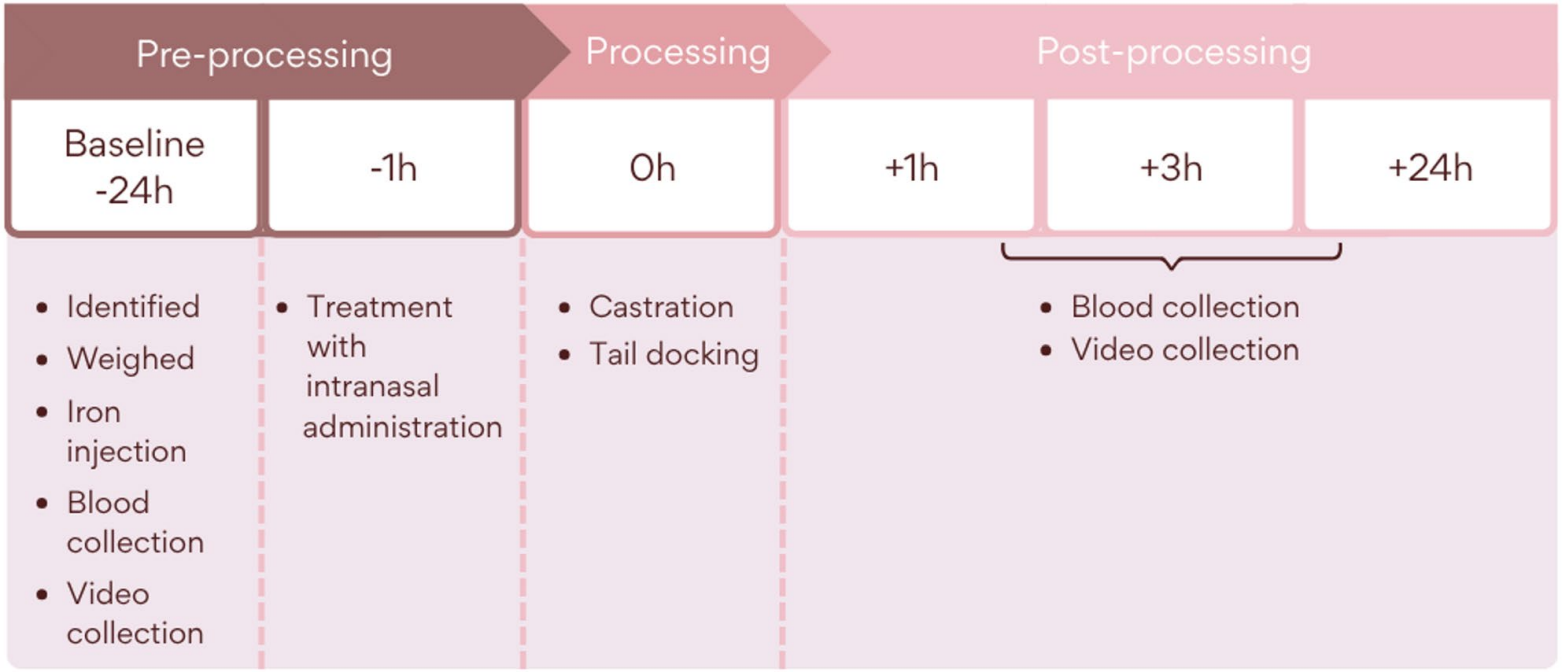
Experimental timeline illustrating pre-processing, processing, and post-processing events. Baseline assessments (− 24 h) included identification, weighing, iron administration, blood sampling, and video recording. Intranasal treatments were administered at − 1 h, followed by castration and tail docking at 0 h. Post-processing blood sampling and video recording were conducted at + 1 h, + 3 h, and + 24 h

A total of 120 Large White x Duroc crossbreed male piglets from 34 litters were enrolled in the study. At processing, piglets had a mean age of 5 days (range: 4–6 days). An overview of the experimental timeline and data collection points is presented in Fig. 1. Although Tmax for intranasal flunixin has been reported between 0.2 and 0.3 h in previous pharmacokinetic studies [12], the + 1 h timepoint was selected to allow for comparison across compounds with potentially different pharmacokinetic profiles. Additionally, available pharmacokinetic data were obtained from 8-week old female pigs, which differ from neonatal male piglets used in this study.

Litters and piglets were enrolled 24 h prior to processing. Eligible piglets received iron supplementation, were weighed and individually identified using permanent markers. Three to four male piglets per litter were included. Piglets were blocked by weight and randomly assigned to one of eight treatment groups:

- F-Sham (*n* = 15): Intranasal flunixin meglumine (Banamine^®^-S injectable solution; Merck Animal Health, Madison, NJ, USA; 2.2 mg/kg); handled but not processed.
- F-Proc (*n* = 15): Intranasal flunixin meglumine (2.2 mg/kg); processed 1 h after drug administration.
- K-Sham (*n* = 15): Intranasal ketoprofen (Ketofen^®^ injectable solution; Zoetis Services LLC, Parsippany, NJ, USA; 4 mg/kg); handled but not processed.
- K-Proc (*n* = 15): Intranasal ketoprofen (4 mg/kg); processed 1 h after drug administration.
- M-Sham (*n* = 15): Intranasal meloxicam (Meloxivet^®^ injectable solution, Dechra Veterinary Products, Overland Park, KS, USA; 0.5 mg/kg); handled but not processed.
- M-Proc (*n* = 15): Intranasal meloxicam (0.5 mg/kg); processed 1 h after drug administration.
- S-Sham (*n* = 15): Intranasal Saline (1 mL); handled but not processed.
- S-Proc (*n* = 15): Intranasal Saline (1 mL); processed 1 h after administration.

Drugs were administered as provided by the manufacturer, without further dilution, 1 h prior to the processing procedure as described by Lopez-Soriano et al. [11]. In short, piglets were held in sternal recumbency, and the assigned treatment was administered into one nostril using a MAD^®^ intranasal mucosal atomization device (Teleflex Incorporated, Wayne, PA, US) attached to a Prima Tech^®^ 0.5 cc bottle mount vaccinator.

### Castration and tail docking procedure

Male piglets assigned to the processing groups underwent surgical castration and tail docking. Castration was performed by making two vertical scrotal incisions using a scalpel blade. Tail docking consisted of removing the proximal two-thirds of the tail using disinfected clippers. All procedures were conducted by a single trained stockperson with over 10 years of on-farm experience. Piglets in sham groups were handled identically to processed pigs but did not undergo any surgical procedures.

### Blood sampling

Blood samples (0.3-3.0 mL) were collected from all enrolled piglets (*n* = 120) using 20G needles, via orbital sinus, as described by Dove et al. [14]. Samples were obtained at baseline (24 h before processing), 1 h, 3 h and 24 h after processing. Blood was collected into lithium heparin tubes and centrifuged within 1 h of collection at 3,500 rpm for 10 min. The resulting lithium heparin plasma was aliquoted into two 1.5 mL microtubes for cortisol analysis and stored at -80° C at the end of each experimental day until analysis.

### Plasma Cortisol quantification

Plasma cortisol concentrations were measured using a commercial enzyme-linked immunosorbent assay (EIA; DetectX Cortisol Kit, Arbor Assays, Ann Arbor, MI, USA). The assay detection limits were 50–3,200 pg/mL. Plasma samples were diluted 1:100 with assay buffer and analyzed in duplicate according to the manufacturer’s instructions.

Absorbance was read at 450 nm using a microplate spectrophotometer (BioTek Epoch, Agilent, Santa Clara, CA, USA). Cortisol concentrations were calculated from a standard curve prepared with kit standards. The mean ± SD intra-assay coefficient of variation for duplicate samples was 6.7 ± 7.5%, and the mean inter-assay coefficient of variation, calculated from two quality-control samples was 10.0 ± 0.1%.

### Behavioral assessment

Thirty-four litters were behaviorally assessed using the Unesp-Botucatu Pig Composite Acute Pain Scale (UPAPS; [10]). Piglets were individually identified using livestock markers by assigning a number on the back, allowing for consistent tracking across timepoints and reliable visual identification during video analysis. Video recordings were obtained in the farrowing stalls at baseline (24 h before processing), 1 h, 3 h, and 24 h post-processing. Each recording lasted 15 min, totaling 480 observation periods (120 piglets x 4 timepoints. Videos were subsequently edited into 4-minute clips, prioritizing periods of activity (avoiding sleeping or nursing periods), as described in the methodology of Lopez-Soriano et al. [9] and Robles et al. [10]. When more than one piglet appeared in the same video, the same segment was used for multiple individuals.

All videos were processed and randomized by one investigator (VRM). A previously trained researcher (CAFM), masked to treatment and hour, evaluated all 4-minute clips. Pain behavior was scored using the UPAPS, which consists of five behavioral items, related to posture, interaction and interest in the surroundings, activity, attention to the affected area, and miscellaneous behaviors [10]. These behavioral items are classified according to four descriptive levels (‘0’, ‘1’, ‘2’, and ‘3’). Level ‘0’ indicates normal behaviors not associated with pain, while levels ‘1’, ‘2’ and ‘3’ indicate pain-altered behaviors, resulting in total scores ranging from 0 (minimum) to 15 (maximum) per piglet. Results are presented as total UPAPS scores.

### Statistical analysis

Cortisol data were analyzed using SAS software (version 9.4; SAS Institute Inc). Multilevel linear mixed models were fitted to analyze repeated measures across hours [baseline (-24 h), 1, 3 h and 24 h post-castration] and treatments groups. Fixed effects included drug, procedure, hour and their interactions, while piglets were included as a random effect to account for repeated measures over time. Model structure was selected based on the lowest Bayesian Information Criterion (BIC). Data were Box-Cox transformed to meet model linearity assumptions. Pairwise comparisons were adjusted using the Tukey-Kramer method.

Behavioral data were analyzed using R software within the RStudio environment (Version 4.3.3; RStudio, Inc., Boston, MA, USA). Functions and packages are presented using the format ‘package::function’.

Modeling was conducted to compare the effects of procedure (Sham vs. Processed), drug (Saline, Flunixin, Ketoprofen, Meloxicam), and hour (Baseline, 1 h, 3 h, 24 h). Overdispersion and excess zeros in UPAPS scores were confirmed using histogram inspection (stats::hist) and the Cameron and Trivedi test (performance::check_overdispersion), justifying the use of zero-inflated models. Zero-inflated models combine logistic and count components within the same modelling framework. A zero-inflated Poisson model (pscl::zeroinfl) provided the best fit compared with alternative models (Poisson, negative binomial, and mixed models with Poisson or negative binomial distributions) based on BIC (stats::BIC).

Total UPAPS scores was used as the response variable, while the three-way interaction among hour, drug and procedures included in the count component (Poisson), and procedure included in the logistic component (Bernoulli). Post hoc multiple comparisons were adjusted using the Bonferroni method (lsmeans::lsmeans and multcomp::cld). For all analyses, statistical significance was set at *P* < 0.05.

## RESULTS

### Plasma cortisol quantification

**Fig. 2:**
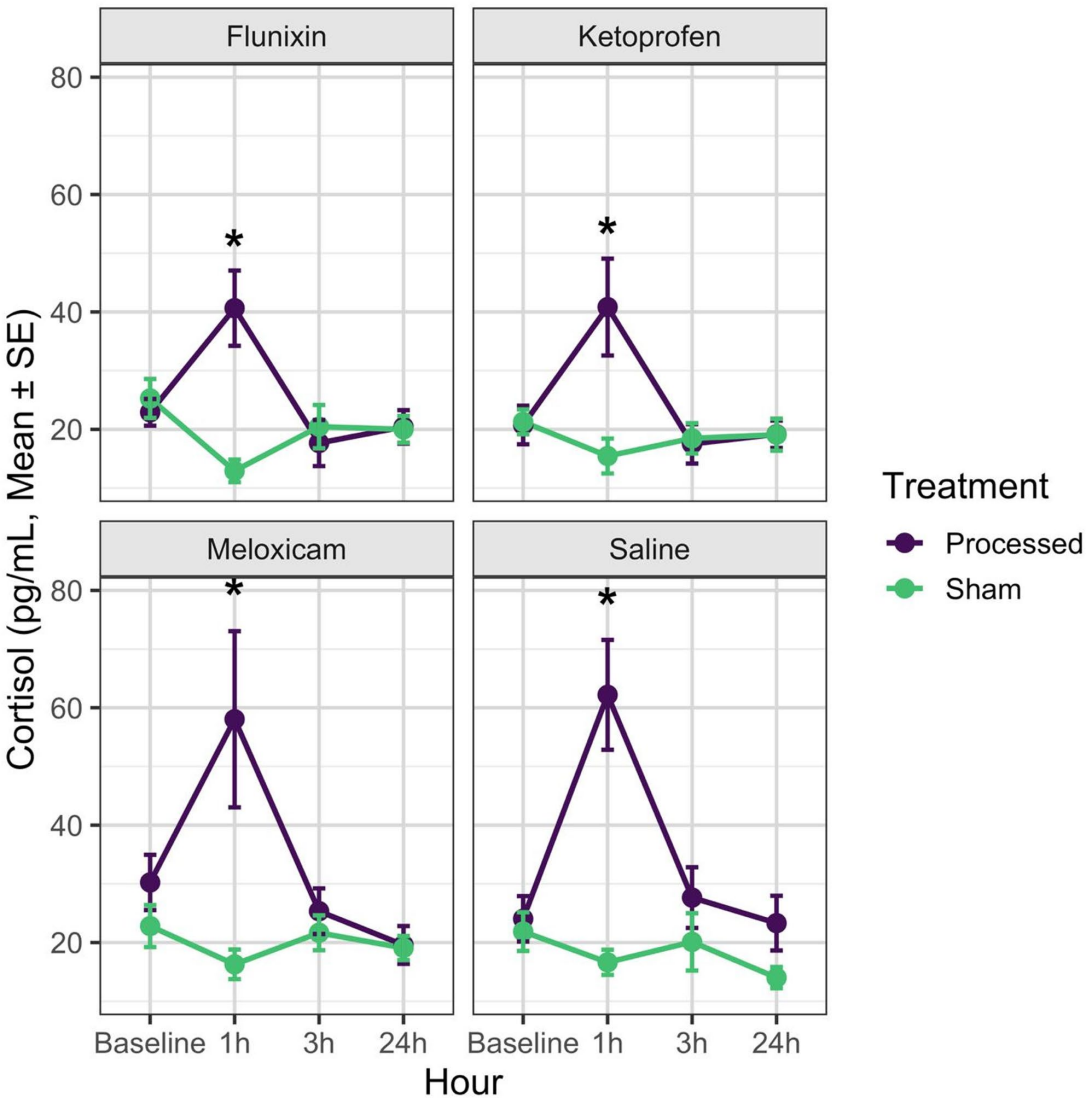
Plasma cortisol concentrations (pg/mL) in sham and processed piglets at baseline and at 1 h, 3 h, and 24 h after the procedure. Values are expressed as mean ± SEM. * Indicates a significant treatment effect (processed vs. sham)

An effect of hour (*P* < 0.0001) and an hour × procedure interaction (*P* < 0.0001) was observed for plasma cortisol concentrations (Fig. 2). At 1 h post-procedure, processed piglets had higher cortisol concentrations than sham piglets. No effect of procedures was detected at baseline, 3 h, or 24 h. No effects of treatment, hour × treatment, or hour × treatment × procedure were detected (*P* > 0.05).

### Behavioral assessment

**Fig. 3:**
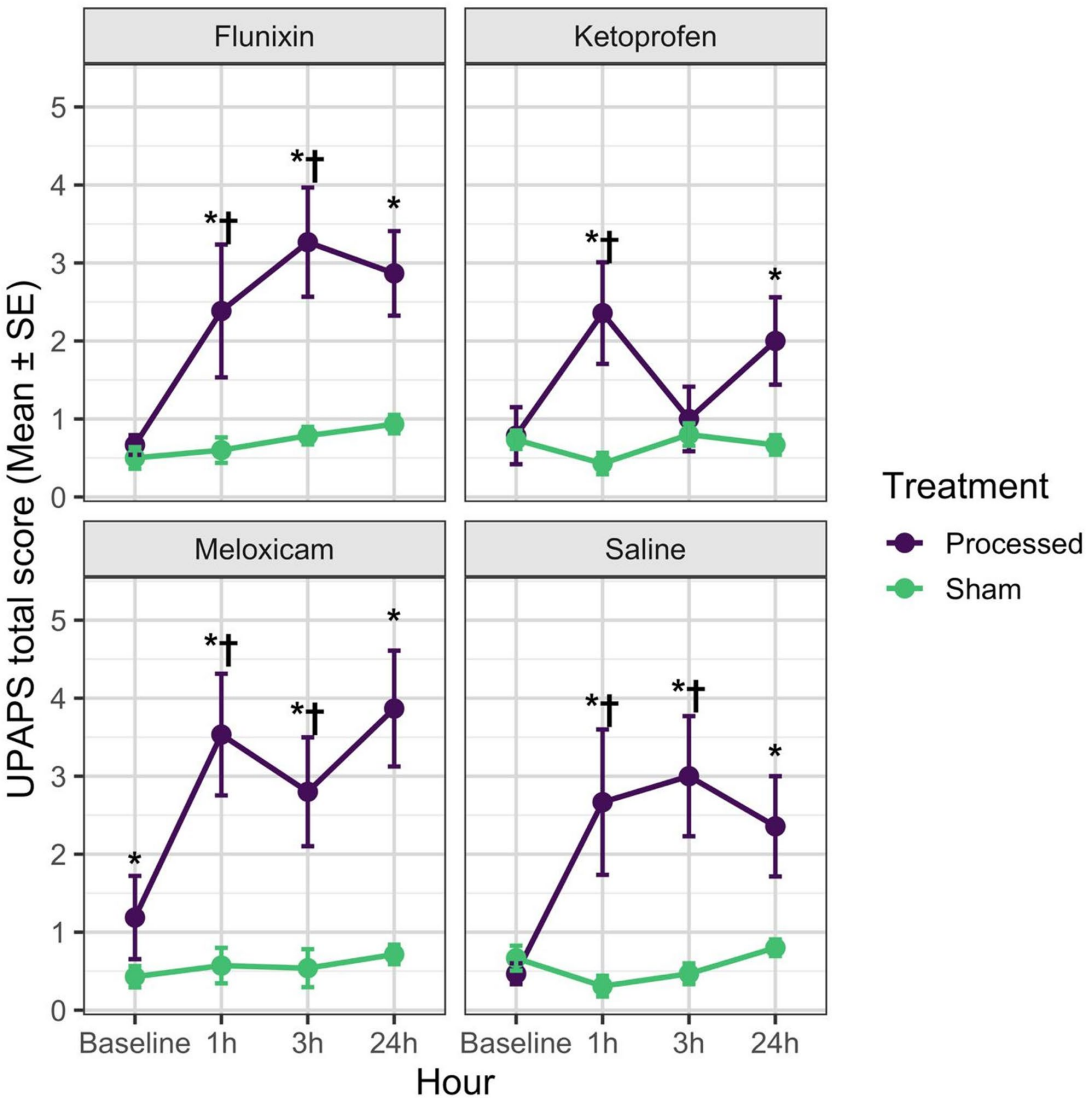
Total UNESP-Botucatu Piglet Acute Pain Scale (UPAPS) scores in sham and processed piglets at baseline and at 1 h, 3 h, and 24 h after the procedure. Values are presented as mean ± SEM. * Indicates a significant treatment effect (processed vs. sham). † Indicates a significant difference from baseline at subsequent time points (1 h, 3 h, or 24 h)

An effect of hour x procedure was observed for UPAPS scores at 1 h (*P* = 0.0001), 3 h (*P* = 0.0002) and 24 h (*P* = 0.0144; Fig. 3) with sham piglets demonstrating lower total UPAPS scores than processed piglets. Total UPAPS scores at baseline were not different when comparing procedure groups (*P* > 0.05).

In addition, a treatment x hour x procedure effect was also observed for total UPAPS scores. Total UPAPS scores increased relative to baseline in processed piglets treated with saline, flunixin and meloxicam 1 and 3 h post procedure (*P* ≤ 0.0001). In contrast, piglets treated with ketoprofen had greater total UPAPS scores at 1 h compared to baseline (*P* < 0.02), but total pain score at 3 h did not differ from baseline (*P* > 0.05). In addition, within the processed group at 3 h, ketoprofen piglets showed lower UPAPS scores than those treated with saline, flunixin, or meloxicam (*P* < 0.05). Processed piglets demonstrated greater UPAPS scores than sham piglets at 1 h and 3 h for saline, flunixin, and meloxicam (*P* ≤ 0.0001). For ketoprofen, this difference occurred at 1 h (*P* = 0.0001) but not at 3 h (*P* > 0.05). At 24 h, the processed group demonstrated greater scores than sham piglets across all treatments (*P* = 0.002 to < 0.0001). At baseline, a difference between procedures was observed only in meloxicam group (*P* = 0.04). An hour or treatment effect within the sham procedure group was not detected (*P* > 0.05).

## Discussion

The present study evaluated the effects of processing and NSAID administration on physiological and behavioral indicators of pain and stress in male piglets. Overall, the results demonstrate that castration and tail docking elicited an acute stress and pain response, evidenced by transient changes in plasma cortisol and sustained increases in UPAPS total score. In contrast, some NSAID treatments modulated pain responses in a drug- and time-dependent manner but did not consistently mitigate pain sensitivity over the 24 h following the procedure.

Processed piglets, with and without analgesic intervention, demonstrated greater cortisol concentrations than sham piglets at 1 h post-procedure, indicating that NSAID treatments did not mitigate the physiological cortisol response. This response is consistent with multiple previous studies showing that painful husbandry procedures, such as castration and tail docking, induce short-term activation of the hypothalamic–pituitary–adrenal (HPA) axis in piglets [2, 3, 15, 16].

Despite the administration of different NSAIDs, no treatment effects were noted on plasma cortisol concentrations across all timepoints. Nixon et al. [3] and Merenda et al. [16] reported rapid increases in cortisol concentrations within the first hour following castration, followed by a gradual decline, indicating that activation of the HPA axis occurs even when pharmacological interventions were applied. Similar patterns have been reported for other NSAIDs, including meloxicam and ketoprofen, with cortisol concentrations returning to baseline within a few hours post-procedure [16, 17], reflecting the short-lived nature of this acute response. Accordingly, the lack of significant differences among NSAID treatments observed in the present study is consistent with published evidence indicating that, although NSAIDs may provide behavioral or inflammatory benefits, their ability to modulate the cortisol response is limited and depends on factors such as drug class, dose, route of administration, and timing relative to the procedure [18].

Behavioral assessment using UPAPS corroborated the presence of procedure-induced pain, as indicated by higher UPAPS total scores at 1 h, 3 h and 24-h post-procedure compared to baseline. This temporal pattern is consistent with previous studies reporting peak behavioral pain expression during the early post-castration period [9, 10]. The persistence of higher UPAPS scores at 24 h post procedure suggests residual discomfort or ongoing tissue sensitivity one day post-procedure. Similarly, other behavioral studies in piglets showed that pain-related behaviors remain elevated for at least 24 h post-castration and are not consistently mitigated by common NSAID treatments [19], highlighting the robustness of behavioral pain responses even when pharmacological modulation is applied. The absence of an hour effect within the sham group confirms that behavioral changes were attributable to the surgical intervention rather than handling or environmental factors.

Within processed piglets, UPAPS total scores differed between drug treatments. Piglets treated with saline, flunixin, and meloxicam showed increased UPAPS total scores at 1 h and 3 h post procedure, indicating incomplete suppression of acute pain-related behaviors. This finding aligns with previous studies demonstrating that NSAIDs administered alone may be insufficient to abolish acute behavioral pain responses following castration [11, 19]. Moreover, another study reported only modest reduction in some pain-related behaviors with meloxicam alone, indicating that NSAIDs may have limited effects on overt behavioral pain indicators unless combined with local anesthetics [20]. These findings align with broader reviews highlighting variability in behavioral outcomes following NSAID analgesia when administered without multimodal approaches (e.g., local anesthesia), underscoring the complexity of interpreting analgesic efficacy solely from behavioral scores [18].

In contrast, ketoprofen-treated piglets exhibited increased UPAPS total scores at 1 h but not at 3 h post-procedure and showed lower scores at 3 h post-procedure compared with other processed groups. This pattern suggests a short-lived attenuation of behavioral pain expression during the intermediate post-procedural period, consistent with previous observations of time-dependent analgesic effects of ketoprofen [3].

Some limitations should be acknowledged when interpreting the results of the present study. Intranasal administration represents a practical, needle-free approach for drug delivery in commercial swine systems, which may facilitate on-farm adoption of analgesic protocols; however, this route may also introduce variability in drug absorption due to factors such as mucosal characteristics and delivery efficiency. In addition, no studies to date have evaluated pharmacokinetic parameters for intranasal administration of ketoprofen or meloxicam, and the only available PK data for intranasal flunixin were generated in 8-week-old female pigs [12]. Although these data suggest similar T_max_ and half-life compared with intramuscular administration, further research is needed to determine pharmacokinetic profiles under neonatal conditions. Additionally, particle size following atomization was not characterized and may influence drug absorption, representing an important area for future investigation.

## Conclusion

Overall, the findings demonstrate that piglet processing results in measurable behavioral and physiological responses. While cortisol reflected a brief activation of the HPA axis, behavioral measures revealed persistent pain-related responses up to 24 h post-procedure. This divergence highlights the complexity of pain expression and the importance of multimodal assessment approaches. The selected physiological and behavioral measures allowed for the evaluation of both acute stress responses and sustained pain-related outcomes. These results further support the need for integrated analgesic strategies, potentially combining NSAIDs with additional local anesthetic modalities, to more effectively mitigate procedural and post-procedural pain.

Non-steroidal anti-inflammatory drugs are more attractive for use in commercial swine systems, as they are not controlled substances and do not require additional training for caretakers compared to intrainguinal or subcutaneous lidocaine. However, despite this practicality, effective pain mitigation at the time of processing was not achieved, and multimodal analgesic protocols are likely needed to ensure adequate pain control both during and after the procedure.

## Data Availability

The data that support the findings of this study are available from the corresponding author upon reasonable request.
